# Supplementary material for: Adaptive evolution in virulence effectors of the rice blast fungus Pyricularia oryzae
Source: PLoS Pathog. 2023 Sep 11;19(9):e1011294. doi: 10.1371/journal.ppat.1011294 (PMC10513199; doi:10.1371/journal.ppat.1011294)
Supplement: S4 Fig — (DOCX) [file ppat.1011294.s013.docx]

**
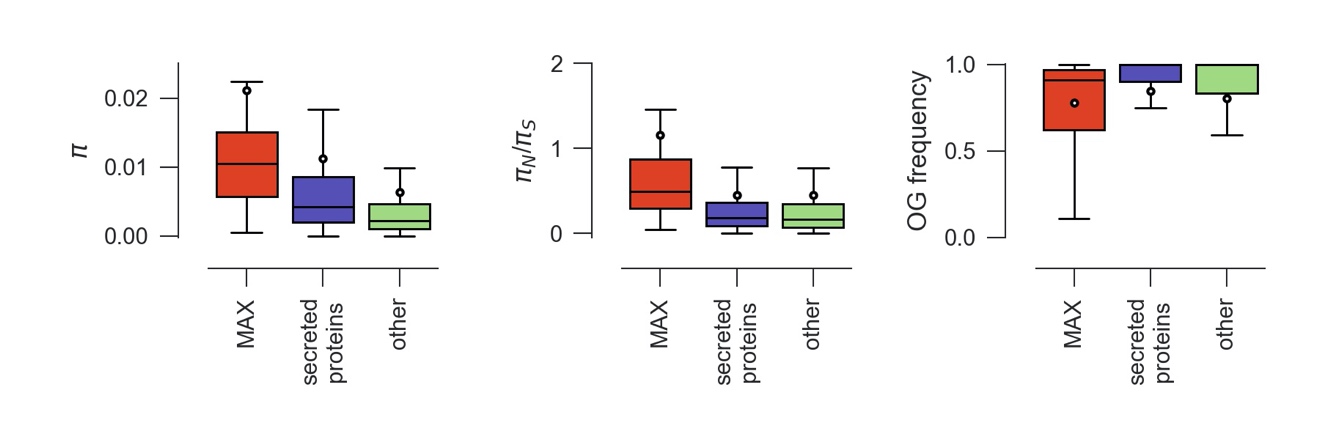
**

S4 Fig. Nucleotide diversity (π), ratio of non-synonymous to synonymous nucleotide diversity (π_N_/π_S_), orthogroup frequency for MAX effectors, other secreted proteins, and other genes. All differences were statistically significant at *p*<0.05 (Kruskal-Wallis tests post-hoc Mann-Whitney U-tests). In box plots, the circle is the mean, the solid black line is the median.
