# Supplementary material for: Adaptive evolution in virulence effectors of the rice blast fungus Pyricularia oryzae
Source: PLoS Pathog. 2023 Sep 11;19(9):e1011294. doi: 10.1371/journal.ppat.1011294 (PMC10513199; doi:10.1371/journal.ppat.1011294)
Supplement: S5 Fig — (DOCX) [file ppat.1011294.s014.docx]

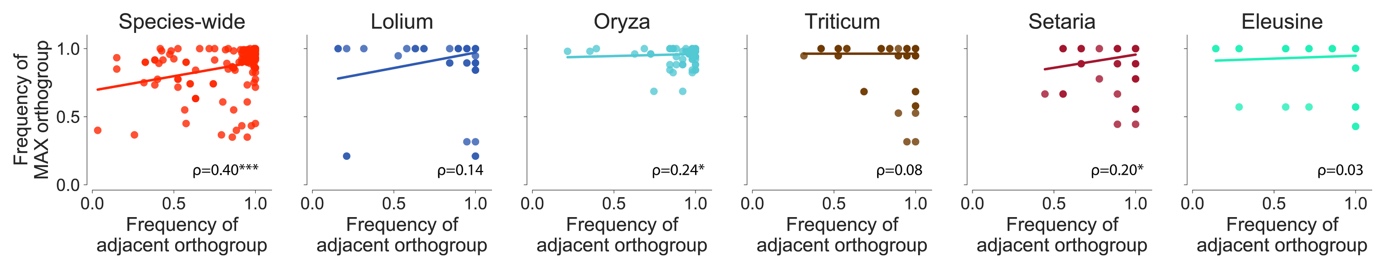


S5 Fig. Frequency of MAX effector orthogroups as a function of the frequency of the adjacent orthogroups in the genome. ρ is Spearman’s rank-order correlation statistic. ****p*<0.001; **p*<0.05.
