# Supplementary material for: Adaptive evolution in virulence effectors of the rice blast fungus Pyricularia oryzae
Source: PLoS Pathog. 2023 Sep 11;19(9):e1011294. doi: 10.1371/journal.ppat.1011294 (PMC10513199; doi:10.1371/journal.ppat.1011294)
Supplement: S8 Fig — (DOCX) [file ppat.1011294.s017.docx]

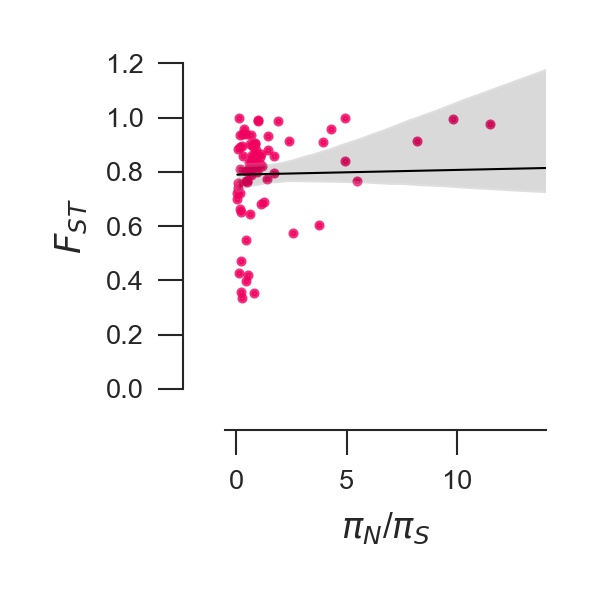


S8 Fig. *F_ST_* versus *π_N_/π_S_* at MAX effectors, with regression model y ~ x and 95% confidence interval for that regression (as estimated using regplot function in Seaborn package with Python3.7).
