# Supplementary material for: Adaptive evolution in virulence effectors of the rice blast fungus Pyricularia oryzae
Source: PLoS Pathog. 2023 Sep 11;19(9):e1011294. doi: 10.1371/journal.ppat.1011294 (PMC10513199; doi:10.1371/journal.ppat.1011294)
Supplement: S1 Text — (PDF) [file ppat.1011294.s023.pdf]

S1 Text. Fitting a generalized linear model to amino acid polymorphism data.

We modeled the probability that an amino acid is polymorphic.

The data used is in S3 Data.txt.

We use the glmer function in R 4.1.2. to fit a binomial regression to our data.

```
library(lme4)
library(boot)
mydata = read.table("input.txt",header=T) # =S3 Data.txt without its first line
mydata$domain <- as.factor(as.vector(mydata$domain))
mydata$accessibility <- as.factor(as.vector(mydata$accessibility))
mydata$feature <- as.factor(as.vector(mydata$feature))
mymodel<-glmer(polymorphic~domain + feature + accessibility + (1|MAX), family=binomial, data=mydata)
summary(mymodel)
```

```
Generalized linear mixed model fit by maximum likelihood (Laplace Approximation) ['glmerMod']
 Family: binomial  ( logit )
Formula: polymorphic ~ domain + feature + accessibility + (1 | MAX)
   Data: mydata

     AIC      BIC   logLik deviance df.resid 
  3469.2   3542.7  -1723.6   3447.2     5899 

Scaled residuals: 
    Min      1Q  Median      3Q     Max 
-1.3210 -0.3352 -0.2478 -0.1719  7.3324 

Random effects:
 Groups Name        Variance Std.Dev.
 MAX    (Intercept) 1.358    1.165   
Number of obs: 5910, groups:  MAX, 91

Fixed effects:
                    Estimate Std. Error z value Pr(>|z|)    
(Intercept)         -2.90333    0.21013 -13.817  < 2e-16 ***
domain2              0.07997    0.15707   0.509   0.6106    
domain3              0.19218    0.16091   1.194   0.2324    
domain4              0.04527    0.16653   0.272   0.7857    
domain5              0.09878    0.16107   0.613   0.5397    
domain6             -0.03777    0.15813  -0.239   0.8112    
featureE             0.03171    0.13590   0.233   0.8155    
featureT            -0.04377    0.13134  -0.333   0.7390    
accessibility30-60   0.29119    0.12006   2.425   0.0153 *  
accessibility60-100  0.58613    0.12602   4.651  3.3e-06 ***
---
Signif. codes:  0 ‘***’ 0.001 ‘**’ 0.01 ‘*’ 0.05 ‘.’ 0.1 ‘ ’ 1

Correlation of Fixed Effects:
            (Intr) doman2 doman3 doman4 doman5 doman6 featrE featrT a30-60
domain2     -0.307                                                        
domain3     -0.292  0.525                                                 
domain4     -0.311  0.499  0.486                                          
domain5     -0.359  0.513  0.503  0.473                                   
domain6     -0.373  0.527  0.513  0.484  0.520                            
featureE    -0.514 -0.078 -0.089 -0.079  0.059  0.071                     
featureT    -0.391 -0.043 -0.069 -0.039 -0.035  0.086  0.638              
accssb30-60 -0.291 -0.174 -0.119 -0.067 -0.093 -0.134  0.141  0.006       
accss60-100 -0.366 -0.105 -0.131 -0.053 -0.070 -0.133  0.307 -0.014  0.573
```

Then, we can estimate prediction intervals for the three subclasses of the factor "accessibility" using the boot package:

```
new.df <- mydata[sample(nrow(mydata), replace = TRUE), ]
new.df$accessibility <- as.factor("0-30")
my.bootstrap.predictions.f <- function(data, indices){
  return(mean(predict(mymodel, newdata = data[indices, ], type = "response", allow.new.levels=TRUE),
na.rm=TRUE))
}
my.boot.obj <- boot(data = new.df[sample(nrow(new.df), 20000, replace = TRUE), ], statistic =
my.bootstrap.predictions.f, R = 2)
quantile(my.boot.obj[[2]], c(0.025, 0.975))
```

```
      2.5%      97.5%
0.08314063 0.08459802
```

```
new.df <- mydata[sample(nrow(mydata), replace = TRUE), ]
new.df$accessibility <- as.factor("30-60")
my.bootstrap.predictions.f <- function(data, indices){
  return(mean(predict(mymodel, newdata = data[indices, ], type = "response", allow.new.levels=TRUE),
na.rm=TRUE))
}
my.boot.obj <- boot(data = new.df[sample(nrow(new.df), 20000, replace = TRUE), ], statistic =
my.bootstrap.predictions.f, R = 2)
quantile(my.boot.obj[[2]], c(0.025, 0.975))
```

```
     2.5%     97.5%
0.1057203 0.1065073
```

```
new.df <- mydata[sample(nrow(mydata), replace = TRUE), ]
new.df$accessibility <- as.factor("60-100")
my.bootstrap.predictions.f <- function(data, indices){
  return(mean(predict(mymodel, newdata = data[indices, ], type = "response", allow.new.levels=TRUE),
na.rm=TRUE))
}
my.boot.obj <- boot(data = new.df[sample(nrow(new.df), 20000, replace = TRUE), ], statistic =
my.bootstrap.predictions.f, R = 2)
quantile(my.boot.obj[[2]], c(0.025, 0.975))
```

```
     2.5%     97.5%
0.1308136 0.1311139
```

We can also compute the observed rates of amino acid polymorphism:

```
newdata <- mydata[ which(mydata$accessibility=="0-30"), ]
mean(newdata$polymorphic)
```

```
[1] 0.08674464
```

```
newdata <- mydata[ which(mydata$accessibility=="30-60"), ]
mean(newdata$polymorphic)
```

```
[1] 0.1052364
```

```
newdata <- mydata[ which(mydata$accessibility=="60-100"), ]
mean(newdata$polymorphic)
```

```
[1] 0.1295611
```
